# Nutritional and ethnomedicinal scenario of koumiss: A concurrent review

**DOI:** 10.1002/fsn3.2595

**Published:** 2021-09-20

**Authors:** Muhammad Afzaal, Farhan Saeed, Fatima Anjum, Numra Waris, Muzzamal Husaain, Ali Ikram, Huda Ateeq, Faqir Muhammad Anjum, Hafiz Suleria

**Affiliations:** ^1^ Department of Food Sciences Government College University Faisalabad Pakistan; ^2^ Department of Dietetics and Nutrition The University of Faisalabad Faisalabad Pakistan; ^3^ Administration Department University of the Gambia Serrekunda Gambia; ^4^ Department of Agriculture and Food Systems The University of Melbourne Australia

**Keywords:** fermented products, koumiss, mare milk, nutritional properties, therapeutic potential

## Abstract

Fermented foods are an essential source of nutrition for the communities living in developing areas of the world. Additionally, traditional fermented products are a rich source of various bioactive components. Experimental research regarding the functional exploration of these products is a way forward for better human health. Among fermented foods, Koumiss is rich in vitamins especially vitamin C and minerals, i.e., phosphorus and calcium. In addition, it is also rich in vitamins A, E, B2, B12, and pantothenic acid. High concentrations of lactose in milk favor bacterial fermentation, as the original cultures decompose it into lactic acid. Koumiss contains essential fatty acids such as linoleic and linolenic acid. Koumiss offers many health benefits including boosting the immune system and maintains blood pressure, good effect on the kidneys, endocrine glands, gut system, liver, and nervous and vascular system. The rich microflora from the fermented product has a pivotal role in maintaining gut health and treating various digestive diseases. The core focus of the current review paper is to highlight the nutritional and therapeutic potential, i.e., anticarcinogenic, hypocholesterolemia effect, antioxidative properties, antibacterial properties, antibacterial spectrum, intestinal enlargement, and β‐galactosidase activity, of Koumiss as a traditional fermented product. Moreover, history and production technology of the Koumiss are also the main part of this review paper.

## Graphical abstract

Fermented commodities are an essential source of nutrition for the communities living in developing areas of the world. Traditional fermented products are a rich source of various bioactive components. Koumiss is rich in vitamins and minerals. It offers many health benefits to people such as boosting the immune system; maintaining blood pressure, good effect on the kidneys, liver, endocrine glands, gut system, nervous and vascular system.

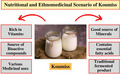

## 1 OVERVIEW

Fermented dairy products have long been known to be a significant component of our nutritious diet, and their therapeutic properties have been recognized since ancient civilization. Evidence suggests that fermented dairy products were produced from about 10,000 BC. Fermentation involves the natural functions of good microbes and their enzymes that can change food into nontoxic foodstuffs with pleasant aroma, taste, texture, and nutrients (Gadaga et al., 1999; Man & Xiang, 2021). A wide range of dairy products is made by fermentation using lactic acid bacteria LAB cultures. Fermentation improves the keeping quality of the product along with enhancement and increasing the digestibility of milk. Several species of lactobacilli are involved in the production of a wide range of conventional dairy products with a wide variety of flavors. There are several products included, such as cheese, pancakes, and porridges. Consumer interest in fermented dairy products is improved because of new food processing processes, changing social attitudes, and evidence of health benefits (Yao et al., 2017).

Koumiss, also known as chigo, chige, arrag, or airag (in Mongolian), a fermented milk product prepared from mare's milk, is famous in Asia, Russia, and Eastern Europe. It is eaten both as a food and an alcoholic beverage (Tang et al., 2020). Koumiss, a traditional Turkish drink, is very famous and popular in Turkish history (it is known as “Turkistan Boza”). The word Koumiss is perhaps arising from a tribe, simply, that lived beside the Kuma River in the grassland of Asia (Robinson et al., 2002). The emergence of Koumiss is closely linked to the nomadic lifestyles of different mammals such as goats, cattle, camels, and mare. Primarily, the objective for the manufacture of Koumiss would be basically to save the vital nutrients in milk. The product is different from dairy products because it is made by using mare`s milk. The first cultures used included many types of LAB and yeast. Traditionally, the origin of the natural environment is the previously boiled Koumiss. In general, Koumiss surpasses two primary fermentation, such as lactic acid fermentation and alcohol fermentation (Chen et al., 2010). Koumiss provides 0.6%–3% alcohol and a little amount of carbon dioxide. Danova et al. (2005) described three types of Koumisses: potent, moderate, and light‐based on lactic acid content (Table 1). The variation in acid content is due to the use of different LAB cultures in the creation of Koumiss. Koumiss is bounded and never marketed worldwide (Malacarne et al., 2002). In the countries where it is generally taken, Koumiss is entertained as a health‐giving product that boosts metabolism and keeps the neurological system and abdominal glands healthy. Commercial production of Koumiss, using cow's milk, which contains higher fat and protein contents but is lesser in lactose than mare's milk, before fermentation, cow's milk is fortified to allow for a similar fermentation.

**TABLE 1:** Types of koumisses

Koumiss types	Lactic acid (%)	pH	Alcohol content (%)
Weak	0.54–0.72	4.5–5	1.0
Moderate	0.73–0.90	3.9–4.5	1.8
Strong	0.91–1.08	3.3–3.9	1.8–2.3

Koumiss is abundant in yeast, trace elements (phosphorus, calcium, magnesium, zinc, iron, manganese, and copper. In addition, the ratio of calcium to phosphorus is 2:1, which is similar to human milk (Ha et al., 2003), antibiotics, rich in vitamins such as ascorbic acid, tocopherols, thiamine, riboflavin, cyanocobalamin, retinol, and vitamin D and also contains many other components, for example, ethyl alcohol, lactic acid and carbonic acid. By Drinking Koumiss we had a therapeutic effect on the body such as on the intestinal tract, metabolism, heart, and abdominal gland; assists the improvement of the immune system; and has been used to handle weight loss and anemic conditions (Guo, Ya, et al., 2019).

The current review article is to highlight the nutritional and therapeutic potential (anticarcinogenic, hypocholesterolemia effect, antioxidative properties, antibacterial properties, antibacterial spectrum, intestinal enlargement, and β‐galactosidase activity) of Koumiss as a traditional fermented product. History, production technology, nutritional profile, Indigenous microflora, and therapeutic potential of the Koumiss are briefly described in the whole article.

## 2 HISTORICAL BACKGROUND

Koumiss is known as an ancient milk‐based beverage. Historical facts prove that the Greeks and Romans often used such drinks in milk. More than 2,500 years ago, guards in southeastern Russia and Scythian tribes in central Asia made Koumiss with mare's milk. William Lubuluqi, a French missionary, came to China in the thirteenth century and gave a comprehensive koumiss to the Mongols. The preparation of Koumiss in China is also reported in history. About 1,500 BC, the household mare was used to make fermented dairy products. Famous Chinese people gained popularity in preparing Koumiss during the Han Yuan Dynasty (202 BC to AD 202) and the Yuan Dynasty (AD 1271–1368). Now, boiled equine milk is widely produced by various names and eaten in several parts of the world principally due to its therapeutic value (Park et al., 2006).

## 3 PRODUCTION TECHNOLOGY

A mare can only be milked as much as it has an uncut calf; Koumiss is generally produced during the short milking time of year, generally from July to October till the summertime (Minjigdorj et al., 2012). The precise amount of milk production differs between individual mares because it depends on the breed, nutrition, atmosphere, and health and supervision status.

Conventionally, fermentation of Koumiss occurs in wood pits or animal skins. After that, urns are employed. For the preparation of Koumiss, two pitchers are left on the outer surface of the yurt and set aside, and half of the hole is covered to a deepness of about 30cm in the land. Afterward, raw filtered mare's milk is added to these pitchers, beaten with a wooden stick, and stored at room temperature (about 20ᵒC) for 1–3 days to multiply the microorganisms. Each day, after extracting the first part of use, a small koumiss aliquot from one of the containers is stored for use as the starter of the next time, and again milk is added to offer further fermentation.

**FIGURE 1:**
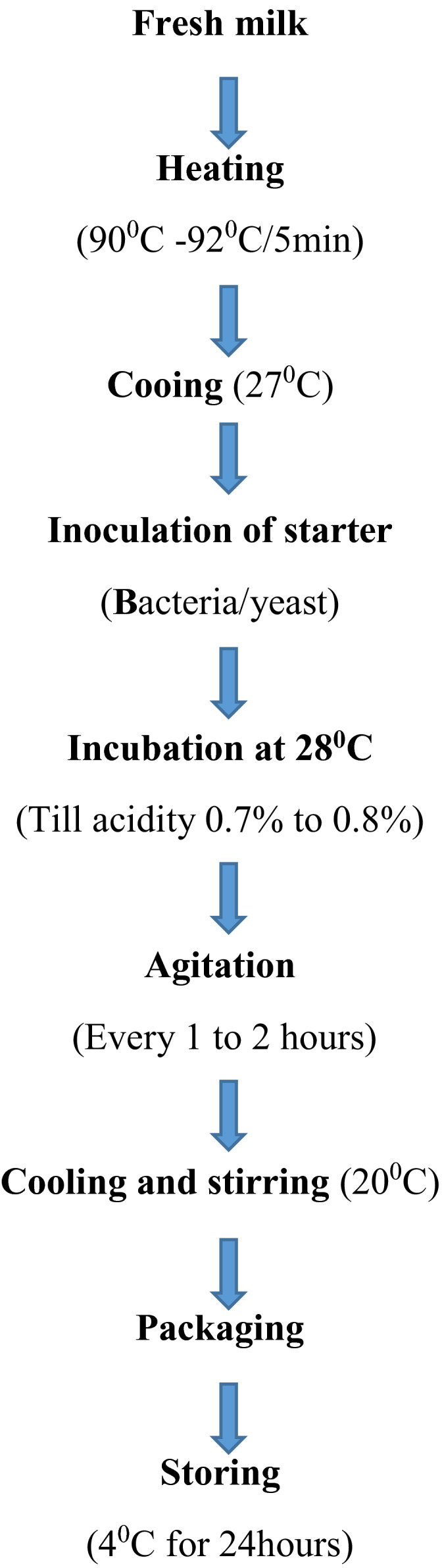
Production technology of Koumiss

In the current production system, in controlled production, to prepare koumiss, mare/cow milk is heated at 90–92℃ for 5–10 min and cooled to 26℃–28℃, and the starter is added. Mare's milk and water are first made from pure thermophilic LAB and yeast cultures. Before being used, LAB and the yeast culture were mixed collectively to create a blended starter culture. The bulk starter culture is added 30% to pasteurized milk. Fermentation is performed at a temperature of about 25ᵒC per 2 hr by stirring constantly. After packing, fermentation continues in bottles at 18ᵒC–20ᵒC for 2–3 hr, then cooled to 4ᵒC–6ᵒC, and then stored until used (Figure 1) (Park et al., 2006).

Pure cultures have been used to produce Koumiss at an industrial level since the 1960s. The microflora of Koumiss principally consists of LAB cultures such as *Lactobacillus bulgaricus*, *Lactobacillus plantarum*, and *Lactobacillus helveticus*, as presented in Table 2. Two types of yeast are also present in Koumiss starter culture, mainly lactose and nonlactose‐fermenting yeast extracted from conventional fermented products. The microflora of Koumiss is closely associated with the source of the product and the environmental situation of the areas from which the product is being made. Koumiss has an effective amount of 5 × 10^7^ CFU ml and 1–2 × 10^7^ CFU ml of bacteria and yeast, respectively. Over storage, the number of bacteria and yeast decreases slowly because of the buildup of lactic acid and ethanol. It can be present in the koumiss microbiota, but its role in odor and taste is minimal. *Acetobacter spp*. is of just little significance. Additional yeast that has been reported to be extracted from conventional Koumiss is *pichia spp*. and *Rhodotorula spp*. (Hou et al., 2019).

**TABLE 2:** Microorganisms present in Koumiss starter

Lactic acid bacteria	Yeasts
*Lb. delbrueckii* ssp. *Bulgaricus*	*Saccharomyces lactis*
*Lb. casei*	*Pichia*
*Str. Lactis* subsp. *Lactis*	*Rhodotorula*
*Lb. lactis* ssp. *Lactis*	*Torula lactis*
*Lb. Leichmanii*	*Saccharomyces lactis*
*Lb. delbrueckii* ssp. *Lactis*	(Lactose‐fermenting)
	*Mycoderma*
	(Nonlactose‐fermenting)
	*Saccharomyces cartilaginosus*
	(Nonlactose‐fermenting)
	*Torula koumiss*
	(Lactose‐fermenting)
*Lactic streptococci*	*Kluyveromyces lactis* or *Kluyveromyces fragilis*
*Lb. acidophilus*	*Kluyveromyces maxianus* var. *maxianus*

Note

Hou et al. (2019).

## 4 NUTRITIONAL COMPOSITION

It contains several substances such as protein, fat, lactose, minerals, enzymes, vitamins, and immune cells, as shown in Table 3. The composition of Koumiss relies on the origin of the milk (mare or cow'), and the other bioactive chemicals can be synthesized using bacterial fermentation. Therefore, in the finished Koumiss, there may be a slight variation due to this different content. For example, mare's milk is richer in lactalbumin, peptone, and other nutrients than cow's and sheep's milk. It is milky green, light, and cold, and has a pungent taste. The digestion of mare's milk is excellent, as it consists of appreciable levels of whey protein. The nutrients of Koumiss are listed below (Lv & Wang, 2009).

**TABLE 3:** Nutritional contents in the final product of Koumiss

Content	Amount (%)
Ash	0.4–0.5
Carbon dioxide	0.5–0.9
Ethanol	0.6–2.5
Lactic acid	0.7–1.8
Lactose	3.5–4.3
Lipid	0.6–1.3
Protein	1.7–1.9
Total solids	10.6–11.3

Koumiss contains all the essential amino acids (especially proline, lysine, tyrosine, valine and leucine) needed by human beings. Thus, Koumiss fulfills all human needs related to amino acids. Koumiss contains a wide variety of minerals, for example phosphorus, calcium, iron, zinc, copper, and manganese. The ratio of phosphorus and calcium is almost the same as human milk (Ha et al., 2003). Koumiss contains high content of vitamin C that provides certain medicinal actions. In addition, it is also rich in vitamins A, B, B2, B12, E, pantothenic acid, and bacteriocins. High concentrations of lactose in milk (6%–7%) favor bacterial fermentation, as the original cultures decompose it into lactic acid, alcohol, and other small molecules. Lactose content in koumiss is 1.4%–4.5%. Koumiss contains essential fatty acids such as linoleic and linolenic acid. In general, mare's milk is loaded with extra fatty acids compared to cow's milk (Orlandi et al., 2003). Such types of fatty acids promote the health of people. The protein composition in Koumiss is 1.7%–2.2% and is based on the milk origin. The ratio of casein to whey is 1:1 near human milk (Ha et al., 2003; Wu et al., 2021).

## 5 THERAPEUTIC POTENTIAL

Koumiss is observed as a good drink in places where it is broadly used. The inclusion of Koumiss enhances various health benefits. Primarily, it is a probiotic‐rich food that humans use. In addition to its probiotic nature, it offers many health advantages, such as antibacterial as well as antifungal characteristics. It regulates the immune system, regulates cholesterol, maintains a healthy digestive system, regulates blood pressure, sugar levels, and produces other essential vitamins.

## 6 ANTIBACTERIAL SPECTRUM

The antagonistic effect of lactic acid antibodies against harmful microorganisms is well illustrated in vitro. For instance, *lactococci* and its capsular substances have been known to reduce the development of *Staphylococcus aureus, Escherichia coli* and certain clostridia. Some of the types of this *Lactococci* are the starters of Koumiss (Chen et al., 2021). Antibiotics are used to treat harmful microorganisms and *E. coli* infections. Despite their helpful actions against pathogenic bacteria, they do some harm to natural flora, resulting in microecological imbalance, physiological dysfunction, and increased vulnerability to external infection (Čižman, 2003, Levy & Marshall, 2004; Tadesse et al., 2012; Dwivedi et al., 2015). As a result, natural medicines, probiotics, and other green products are receiving greater attention as alternatives to antibiotics. Koumiss is a fermented mare's milk that has been shown to help with cardiovascular illness, TB, and diarrhoea by nourishing arteries, alleviating poor moods, and improving digestion (Wu et al., 2009). Yeasts are the most common microorganisms in koumiss, and they play a vital part in the fermentation process as well as providing medicinal benefits. Some yeasts have been found to have antibacterial effects on E. coli, potentially through the production of antibacterial chemicals such killer toxins and organic acids in metabolism (Etienne‐Mesmin et al., 2011). We identified *Kluyveromyces marxianus* and *Saccharomyces cerevisiae* from koumiss and showed that their antibacterial components were effective against *E. coli* O8 (Chen et al., 2017, 2019). In conclusion, four antibacterial compounds derived from koumiss yeasts had better antibacterial effects against three Gram‐negative, three Gram‐positive bacteria, and five E. coli strains, indicating that they had a broad antibacterial spectrum and could be used as broad‐spectrum antibacterial agents.

## 7 ANTICANCER POTENTIAL

Malignancy is one of the chief causes of death around the globe. It is the uncontrolled development of abnormal cells in the body, such as a lump. Koumiss probiotics are believed to inhibit tumor growth by inhibiting carcinogenic chemicals and increasing the immune system (Leite et al., 2013). Fermented foods are referred to as “naturally fortified functional nutrients” because these aid in the maintenance of a healthy gut microbiota, which protects against illness and physiological balance (Sharma et al., 2018). LAB play a key role in these processes because they produce secondary metabolites such as bacteriocins, ethanol, acetic acid, aroma compounds, exopolysaccharides, bioactive peptides, vitamins, and some enzymes during fermentation (Leroy & De Vuyst, 2004; Stanton et al., 2005). The anticancer, antioxidant, and other properties of the produced bioactive peptides have a favorable impact on health. *(*Rong et al., 2019) discovered that bacteria *Lactobacillus helveticus* NS8 present in koumiss culture are effective against colorectal cancer in vitro, causing enterocyte growth inhibition, apoptosis induction, significant suppression of NF‐B activation, and rearrangement of antiinflammatory cytokine IL‐10. Koumiss probiotics are thought to slow tumor growth by preventing the production of carcinogenic compounds and boosting the immune system (Leite et al., 2013).

## 8 GASTROINTESTINAL PROLIFERATION

Koumiss is easily absorbed and shows a higher concentration of digestion than whole milk (digestion of whole milk is 32% compared to fermented milk 91% per hour). The health‐promoting bacteria present in Koumiss produce enzymes that can digest food in the gut and, as a result, make nutrients easier to absorb. In addition, these bacteria produce specific vitamins, which make food richer in nutrients. The better digestion of Koumiss is due to partial peptonization and secretion of ferment by glands of the digestive tract. It stimulates the appetite for their delicious, refreshing, and sharp taste and enhances the functioning of the CNS. It increases the secretion of juices in the stomach, retaining the desired amount of calcium and phosphorus, leading to increased digestive capacity (Ya et al., 2008).

## 9 IMMUNE SYSTEM BOOSTER

A daily diet of Koumiss improves the immune system, as about 80% of the body's tissues are concentrated in the intestines. Immune system is significantly weakened owing to the removal of intestinal bacteria. It is confirmed that gut bacteria‐free animals had low levels of essential white blood cells and other protective chemicals in their blood. When naturally occurring, bacteria are reintroduced into the animal's intestinal tract, white blood cells are activated, and the immune system is strengthened. Bacteria from fermented foods naturally produce chemicals that pass through the intestinal wall and stimulate the formation of immune cells in the immune system (Kondybayev et al., 2021). The effects of Koumiss on the immune system and its endorsement of antibacterial activities have been illustrated. The results showed that it could appreciably enhance the immune system of experimental animals. Fresh mare milk can boost the thymus and spleen index and build up the functions of macrophages and amplify the ratio of hemolysin in blood serum. In addition, fresh mare milk increases the weight of the immune organs of rats and boost up the normal immune functions, regulates cell immune capabilities, and controls unusual body fluid immune systems (Ya et al., 2008).

## 10 EFFECT OF KOUMISS ON BLOOD PRESSURE

High blood pressure, a problem in today's world, is determined by many factors, most notably the genetic makeup and the environment. Those already suffering from abnormal blood pressure are at serious risk of evolving other life‐threatening illnesses, such as heart diseases and stroke. Many researchers have successfully established the positive effect of milk intake on systolic pressure than calcium can have on its own. Regular consumption of dairy products provides three important antihypertensive nutrients (calcium, whey‐derived peptides, and casein phosphopeptides) and other active peptides that lower blood pressure (Li et al., 2017).

## 11 HYPOCHOLESTEROLEMIC AND ANTIINFLAMMATORY PROSPECTIVE

The World Health Organization (WHO) has foreseen that by the year 2030, heart disease will stay a primary reason for death, touching about 23.6 million people. One of the chief threats for CHD is hypercholesterolemia (Fradi et al., 2008). The greater the total serum cholesterol, the higher the danger of suffering from CHD. Although therapies efficiently reduce cholesterol levels, they are costly and can have severe aftereffects (Bliznakov, 2002). There is a need to focus on scientific studies of natural food products that can significantly lower serum cholesterol levels with little or no side effects. In recent years, the probiotic potential LAB cultures have been evaluated. One of the positive effects of probiotic‐related food is its capability to lower serum cholesterol levels (Lye et al., 2010).

There is an ongoing interest in developing active natural ingredients from food (e.g., certain traditional dairy products or traditional dairy products supplemented with Lactobacillus acidophilus such as Koumiss) that can reduce serum cholesterol concentration (Tortuero et al., 1975).

## 12 ANTIOXIDANT POTENTIAL

Oxidative stress is linked with a lot of diseases. It has been known that thiobarbituric acid‐reactive substances, which are used as a sign of lipid peroxidation, increase in the body of diabetic patients and heart disease (Li et al., 2013). For that reason, the outcome of *lactobacilli* on lipid peroxidation inhibition was studied. Since several portions of the LAB are rooted within the duodenum, crucial to the discharge of intracellular contents, the suppressive effects of intracellular cell‐free extracts on lipid peroxidation have also been examined.

Weak cells and internal CFE in all tested strains show antioxidative activity. However, intracellular CFE had a much higher rate of suppression of linoleic acid peroxidation than weak lactobacilli cells extracted from koumiss samples. More muscular cells are more expected to enter the bloodstream, therefore only preventing intestinal peroxidation. The fact that intracellular CFE has a better suppressive effect in contrast with unchanging cells proposes that the extent of antioxidant activity in intracellular CFE was superior to the time provided to the media by weak cells. Therefore, the antioxidative effect of intracellular CFE in humans generally relies on the quantity of intracellular discharge secreted into the small intestine into the bloodstream.

## 13 Β‐GALACTOSIDASE ACTIVITY

LAB such as *Lacticaseibacillus casei* and yeast in Koumiss may bear a lower level of acidity and are responsible for the change of lactose into lactic acid with the assist of its β‐galactosidase enzyme. The critical function of LAB culture in Koumiss is to use lactose as a substrate and change to lactic acid through fermentation. Lactose is considered a free sugar and is broken down with β‐galactosidase into glucose and galactose. Both of these products are synthesized at the same time, using glycolytic and D‐tagatose 6‐phosphate methods correspondingly. This is particularly beneficial for people that cannot absorb lactose due to a lack of the enzyme lactase (Singh et al., 2017).

## 14 PREVENTION OF APOPTOSIS

Cell contraction, nuclear dissociation, and chromatin depletion are incorporated in a series of general behavioral apoptosis (Saraste & Pulkki, 2000). LAB cultures are widely recognized as a type of probiotic that has many health benefits for humans. However, recent research has shown that the biological activity of LAB can be obtained through live or dead bacteria (Gonet‐Surowka et al., 2007). Exopolysaccharides (EPS) produced by LAB have specifically found their potential interests, such as essential chemicals and health services (Laiño et al., 2016). EPS is usually associated with all types of polysaccharides present externally to the microbial cell wall. In addition, EPS characterize one of the significant vital properties of the LAB product (Ruas‐Madiedo et al., 2002), which are reported to have many physical functions. Among them, column‐fighting activities have yielded high interest rates due to the increasing amount and higher death rate of cancer patients. Although antitumor agents are currently used to manufacture highly active chemicals, many uncertainties have been increased about their safety and undesirable outcomes (Ehrke, 2003). That public care has been a move to recognizing antitumor substances from natural sources (Yang et al., 2013). EPS from LAB can be provided as a substitute for antidepressant drugs from safe natural sources examine by a large number of researchers (Deepak et al., 2016; Górska‐Frączek et al., 2013; Liu et al., 2011). The current study examined the effects of EPS from the nine formerly reported components of Lactobacillus with increased levels of bioactivity in HT‐29 cells used in numerous EPS antitumor activity studies (Park et al., 2007; Wang et al., 2015).

## 15 FUTURE PERSPECTIVES

The most common method to find out the microbiota of fermented foods has the constant use of conventional media. Though, all microorganisms cannot be simply cultivated, therefore, continuous cultural media improvements have been made such as in situ growth (Guo, Ya, et al., 2019). Hence, culturally representative methods are significant to examine the metagenomic formation of varied and composite microbial populations of substandard food products. When DNA sequencing is joined with bioinformatics, the employment and characteristics of the microorganisms are quickly and precisely strain specifically characterized. For instance, the use of different techniques such as metabarcoding and metagenome shotgun sequencing to identify viruses and harmful substances in fermented food products (Jagadeesan et al., 2019). Meta transcriptomics is another technique that is presently considered a choice to perform in‐depth research into the genetic makeup of organic foods of microbial populations. The outcome can be constant with other methods developed for “Omics,” such as proteomics and metabolomics, to gain an in‐depth understanding of the relations of microorganisms to the sensory and physicochemical features of fermented food products. Without these processes, single‐cell growth microbiome analysis techniques are not expected. They should in the future show the rare bacteria that are fermented in food parts and therefore be used to control and altered dietary microbiomes (Yao et al., 2017).

Furthermore, fermentation of healthy beverages needs meticulous supervision in order to reach consistency, sensory, and safety standards. Alteration in the concentration of sugar and other complex requires careful consideration during and after fermentation and is especially essential with look upon to the production of ethanol and carbon dioxide.

## 16 CONCLUSION

Fermented products are the important part in the human diet. Koumiss is traditional fermented product with high nutritional profile and functional characteristics. It has many therapeutical properties including antioxidant, antiinflammatory, anticancer antibacterial as well as antifungal potential. It is positively playing role in gastrointestinal proliferation, boost immune system, maintain blood pressure, and prevent the apoptosis.

## 17 STUDIES INVOLVING HUMAN SUBJECTS

This study does not involve any human testing.

## 18 STUDIES INVOLVING ANIMAL OR HUMAN SUBJECTS

This study does not involve any human or animal testing.

## CONFLICT OF INTEREST

The authors declare that they do not have any conflict of interest.

## AUTHOR CONTRIBUTIONS

**Muhammad Afzaal:** Writing‐original draft (equal). **Farhan Saeed:** Supervision (equal). **Fatima Anjum:** Writing‐review & editing (equal). **Numra Waris:** Data curation (equal). **Muzzamal Hussain:** Investigation (equal); Software (equal); Validation (equal). **ALI IKRAM:** Writing‐review & editing (equal). **Huda Ateeq:** Resources (equal); Visualization (equal). **Faqir Muhammad Anjum:** Conceptualization (equal); Supervision (equal). **Hafiz Ansar Rasul Suleria:** Data curation (equal); Visualization (equal).

## ETHICAL APPROVAL

This study does not involve any human or animal testing.
